# Increased macrophages and changed brain endothelial cell gene expression in the frontal cortex of people with schizophrenia displaying inflammation

**DOI:** 10.1038/s41380-018-0235-x

**Published:** 2018-09-13

**Authors:** Helen Q. Cai, Vibeke S. Catts, Maree J. Webster, Cherrie Galletly, Dennis Liu, Maryanne O’Donnell, Thomas W Weickert, Cynthia Shannon Weickert

**Affiliations:** 1 Schizophrenia Research Laboratory, Neuroscience Research Australia (NeuRA), Sydney, NSW Australia; 2 School of Psychiatry, University of New South Wales, Sydney, NSW Australia; 3 Stanley Medical Research Institute, Kensington, MD USA; 4 Discipline of Psychiatry, School of Medicine, The University of Adelaide, Adelaide, SA Australia; 5 Northern Adelaide Local Health Network, Adelaide, SA Australia; 6 Ramsay Health Care (SA) Mental Health Services, Adelaide, SA Australia; 7 Department of Neuroscience and Physiology, Upstate Medical University, Syracuse, NY USA

**Keywords:** Molecular biology, Neuroscience

## Abstract

Elevated pro-inflammatory cytokines exist in both blood and brain of people with schizophrenia but how this affects molecular indices of the blood–brain barrier (BBB) is unclear. Eight mRNAs relating to BBB function, a microglia and three immune cell markers were measured by qPCR in the prefrontal cortex from 37 people with schizophrenia/schizoaffective disorder and 37 matched controls. This cohort was previously grouped into “high inflammation” and “low inflammation” subgroups based on cortical inflammatory-related transcripts. Soluble intercellular adhesion molecule-1 (sICAM1) was measured in the plasma of 78 patients with schizophrenia/schizoaffective disorder and 73 healthy controls. We found that sICAM1 was significantly elevated in schizophrenia. An efflux transporter, ABCG2, was lower, while mRNAs encoding VE-cadherin and ICAM1 were higher in schizophrenia brain. The “high inflammation” schizophrenia subgroup had lower ABCG2 and higher ICAM1, VE-cadherin, occludin and interferon-induced transmembrane protein mRNAs compared to both “low inflammation” schizophrenia and “low inflammation” control subgroups. ICAM1 immunohistochemistry showed enrichment in brain endothelium regardless of diagnosis and was localised to astrocytes in some brains. Microglia mRNA was not altered in schizophrenia nor did it correlate with ICAM1 expression. Immune cell mRNAs were elevated in “high inflammation” schizophrenia compared to both “low inflammation” schizophrenia and controls. CD163+ perivascular macrophages were identified by immunohistochemistry in brain parenchyma in over 40% of “high inflammation” schizophrenia brains. People with high levels of cytokine expression and schizophrenia display changes consistent with greater immune cell transmigration into brain via increased ICAM1, which could contribute to other neuropathological changes found in this subgroup of people.

## Introduction

Schizophrenia is a heterogeneous disorder with unknown aetiology, however dysregulation of the immune system has often been identified and is increasingly perceived as an important factor in disease pathogenesis [1–7]. Prenatal infection and increased levels of cytokines in mothers during pregnancy increases the risk of schizophrenia in the offspring [8, 9]. Elevated levels of proinflammatory cytokines are found in people with schizophrenia and cytokine increases appear exaggerated in a substantial subgroup of patients [3, 10–14].

Pro-inflammatory cytokine mRNAs are elevated in the frontal cortex of people with schizophrenia. We find ~38% of schizophrenia cases have a significant increase in mRNA levels of interleukin *(IL)-1β, IL-6, IL-8* and *SERPINA3* in both the dorsolateral prefrontal cortex (DLPFC) and the orbitofrontal cortex (OFC) [14, 15]. Volk et al. also report elevated immune-related transcripts including *IL-1β*, *IL-6* and *IL-8* in the prefrontal cortex in schizophrenia [16]. Using a similar stratification method to that used in brain, blood cytokine mRNAs can be used to classify a “high and low inflammatory biotype” in people living with schizophrenia. Indeed, around 40% of living patients with schizophrenia can be classified as having a “high inflammatory biotype” based on peripheral blood samples, and those individuals have greater impairments in language and a significant reduction in cortical grey matter volume compared to “low inflammatory biotype” patients [13]. When human brain tissue is measured more directly with stereological techniques, a greater reduction in cortical grey matter volume is also found in schizophrenia cases with elevated brain cytokines compared to controls and schizophrenia cases without an elevation in brain cytokines [15]. However, the cellular source of elevated cortical cytokine mRNAs has not been clearly identified in people with schizophrenia. The blood–brain barrier (BBB) may be involved because inflammatory-related transcripts have been found to be dysregulated specifically in endothelial cells in postmortem schizophrenia brains [17].

From the perspective of a neuroinflammatory hypothesis of schizophrenia [18], the brain vasculature may be affected by chronic and/or unresolved inflammation [19, 20]. There is an up-regulation in expression of brain endothelial interferon-induced transmembrane protein (IFITM) [4, 16, 21], a viral restriction factor, in schizophrenia. During inflammation in general, adhesion molecules such as vascular cell adhesion molecule-1 (VCAM1) and intercellular adhesion molecule-1 (ICAM1) are significantly elevated in the endothelium [22, 23] to allow attachment and transmigration of leukocytes across the BBB [24–27]. Peripheral measures of soluble ICAM1 (sICAM1) and VCAM1 have been reported to be both decreased [28, 29] and increased [30–32] in schizophrenia dependent on the stage of illness and medication status. To our knowledge, there have been no measurements of adhesion molecule expression in brains from people with schizophrenia and limited exploration of evidence to support the hypothesis that there may be peripheral immune cell infiltration [33, 34].

Not only could molecules involved in the transmigration of immune cells into the brain be altered in schizophrenia, but so could molecules involved in other transcytotic pathways. Tight junction and adherens junction proteins such as occludin (OCLN) and VE-cadherin (CDH5) regulate the adherence of endothelial cells to one another and this affects the movement of substances across the BBB [35]. Efflux transporters traffic unwanted molecules and endotoxins from the brain across endothelial cells of the BBB. The ATP-binding cassette transporters are cell-membrane efflux transporters that include P-glycoprotein (ABCB1), breast cancer resistance protein (ABCG2) and multidrug resistance protein (ABCC1). These transporters have varying affinities towards a wide range of substrates and prevent accumulation of damaging exogenous compounds in the brain [36]. We hypothesised that the expression of endothelial cell specific molecules and molecules involved in trafficking of cells may be different in the prefrontal cortex of people with schizophrenia vs. controls.

Elevated cytokines may exaggerate possible changes in endothelial cells and increase infiltration of white blood cells, particularly as IL-8 is a chemoattractant for monocytes [37]. Thus, we aimed to determine whether changes in mRNA expression of proteins associated with the BBB and with immune cell populations could be detected in individuals with schizophrenia compared to controls and further, the degree to which proportions of these same factors may be altered according to previously described “high” or “low inflammation” biotypes (formed on the basis of expression of cortical inflammatory markers) [14, 15]. We hypothesised gene expression changes would be consistent with alterations in a breakdown of transport function and barrier function in schizophrenia, and that this would be more pronounced in the subgroup of people with schizophrenia with increased cortical inflammation. To assess whether cytokines or antipsychotic treatment are potential contributors to gene expression changes, we also measured the effects of IL-1β and antipsychotics on mRNA expression in cultured human endothelial cells. Furthermore, as an initial step towards translating our research to the clinic, we determined whether ICAM1 is elevated in the periphery by measuring its soluble form in plasma in an independent cohort of living patients with schizophrenia or schizoaffective disorder and healthy controls.

## Materials and methods

### Human postmortem brain tissue

DLPFC (BA46, *n* = 74) and OFC (BA11, *n* = 76) tissue from individuals with schizophrenia and controls was obtained from the NSW Brain Tissue Resource Centre (demographics, Table 1). There was a 90% overlap (*n* = 71) between DLPFC and OFC cases. Diagnostic groups were matched for age, brain pH, RNA integrity number (RIN) and postmortem interval (PIM). The brains collected by the NSW Brain Tissue Resource Centre undergo neuropathological examination including screening and exclusion of cases with neurodegenerative, infectious or cerebrovascular disease or microscopic evidence of cerebral hypoxia. A sample size of 74 is expected to have >80% power to detect a change with a sensitive effect size (*d* = 0.253). The use of brain tissue for this study was conducted in accordance with the latest version of the Declaration of Helsinki, after review by the Human Research Ethics Committee at the University of New South Wales (HREC #12435).

**Table 1:** Demographics of the schizophrenia and matched unaffected control subjects in the New South Wales Brain Tissue Resource Centre postmortem cohort

Demographics	DLPFC cohort		OFC cohort	
	Control (*n* = 37)	Schizophrenia (*n* = 37)	Control (*n* = 38)	Schizophrenia (*n* = 38)
Age in years ± s.d	51.14 ± 14.62	51.32 ± 14.13	52.55 ± 14.51	52.24 ± 14.52
Gender	7F:30M	13F:24M	10F:28M	13F:25M
Hemisphere	23R:14L	17R:20L	24R:14L	19R:19L
Brain pH ± s.d.	6.66 ± 0.29	6.61 ± 0.30	6.68 ± 0.27	6.61 ± 0.30
PMI (hours) ± s.d.	24.80 ± 10.97	28.46 ± 13.77	26.43 ± 11.69	28.21 ± 13.57
Months of freezer storage ± s.d.	69.62 ± 42.71	78.89 ± 37.24	69.57 ± 42.73	79.80 ± 36.83
RIN ± s.d.	7.30 ± 0.57	7.27 ± 0.58	7.59 ± 0.83	7.51 ± 0.84
Age (years) at onset ± s.d.	–	23.70 ± 6.10	–	23.71 ± 6.21
Duration of illness (years) ± s.d.	–	27.62 ± 13.82	–	27.58 ± 14.06
Chlorpromazine mean equivalent dose (mg) ± s.d.	–	691.64 ± 502.20	–	677.14 ± 505.6

*DLPFC* dorsolateral prefrontal cortex, *OFC* orbital frontal cortex, *SD* standard deviation, *PMI* postmortem interval, *RIN* RNA integrity number, *F* female, *M* male, *R* right, *L* left, *mg* milligrams

### Quantitative PCR of postmortem tissue

RNA was extracted with TRIzol (Thermo Fisher, Carlsbad, CA, USA) from tissue homogenates. SuperScript First-Strand Synthesis kit (Thermo Fisher) was used to generate cDNA as described previously [38]. In the DLPFC, eight brain endothelial cell related transcripts were chosen to represent transporters (*ABCB1, ABCC1, ABCG2*), tight junction proteins (*OCLN, CDH5*), adhesion molecules (*ICAM1, VCAM1*) and an immune related molecule (*IFITM)*. The *IFITM* probe assay was designed to detect three *IFITM* transcripts: *IFITM1*, *IFITM2*, and *IFITM3*. Transcripts of a microglia marker (IBA1) and three peripheral immune cell surface markers were also measured (*CD163*: perivascular macrophages*, CD14*: monocytes*, FCGR3A (CD16)*: natural killer cells and activated macrophages/monocytes). *ICAM1* mRNA expression was also measured in the OFC. All mRNAs were measured by qPCR on ABI Prism 7900HT system using Taqman Gene Expression Assays (Supplementary Table 1). The averaged raw data from triplicate measurements were normalised to the geometric mean of four housekeeper control mRNAs (β-actin, TATA box-binding protein, ubiquitin C, and GAPDH), which did not differ significantly in their expression between diagnostic groups [38].

### Immunohistochemistry

There is high overlap between the OFC and DLPFC cases with 80% (*n* = 57) of cases assigned to the same “high/low inflammatory” subgroup following clustering using cortical cytokine mRNA as determined in previous publications [14, 15]. Fresh frozen OFC tissue sections were cryostat sectioned (14 µm). Immunofluorescence was used to co-localise a brain endothelial marker (collagen IV) with ICAM1 in all cases. A subgroup of cases were also analysed for astrocyte (GFAP) and ICAM1 co-localisation (*n* = 20). We used 3,3-Diaminobenzidine immunohistochemistry to analyse CD163 localisation in all cases. Tissues from a subgroup of cases were double-labelled with collagen IV and CD163 antibodies (*n* = 5). Antibody details are presented in Supplementary Table 2. Negative control slides were included in all experiments by excluding primary antibodies (Supplementary Fig. 1).

For double-label immunofluorescence, OFC tissue sections were fixed in 4% paraformaldehyde (Sigma-Aldrich. St Louis, MO, USA) for 10 min at 4 °C and blocked for 60 min in 10% normal goat serum (Millipore, Temecula, CA, USA), 10% normal donkey serum (Millipore) and 0.3% Triton X-100 (Sigma-Aldrich) in PBS for 60 min. Slides were incubated with primary antibodies overnight at 4 °C. After washing, secondary Alexa Fluor antibodies were added for 1 h. Slides were washed in 15 mM cupric sulphate (Sigma-Aldrich) and 50 mM ammonium acetate (Sigma-Aldrich) for 2 × 15 min to minimise autofluorescence. Slides were counterstained with 1:1000 DAPI (Sigma-Aldrich) in PBS and cover-slipped with anti-fade mounting media (Citifluor AF1, London, UK).

For 3,3-Diaminobenzidine immunohistochemistry, tissue sections were fixed in 4% paraformaldehyde (Sigma-Aldrich) for 10 min at 4 °C and submerged in 3:1 100% methanol in 3% H_2_O_2_ for 20 min to block endogenous peroxidases. Anti-CD163 primary antibody was applied overnight at 4 °C. The next day, slides were washed and incubated with horse anti-mouse IgG biotinylated secondary antibody for 1 h. Slides were washed and incubated with avidin-biotin-peroxidase complex (Vectastain ABC kit; Vector Laboratories) for 1 h, treated with 3,3-Diaminobenzidine for ~4 min (Sigma, Castle Hill, NSW, Australia), washed, counterstained with Nissl and coverslipped.

Images were captured using a Nikon Eclipse 90i laser-scanning microscope (Nikon D-Eclipse C1, Nikon Australia, Rhodes, NSW, AUS) and a Zeiss Axio Imager 2 microscope (Zeiss Australia, Lonsdale, SA, AUS). Immunoreactivity was assessed by microscopically scanning an entire bank of the gyrus rectus at ×40 magnification. The researcher was blind to diagnosis and inflammatory status, which was *a priori* determined based on cytokine and SERPINA3 mRNA levels using clustering [15]. Images were processed using ImageJ (v1.50e, NIH, Bethesda, MD, USA).

### Human endothelial cell culture

hCMEC/D3 is an immortalised human brain microvascular endothelial cell line derived from human temporal lobe microvessels isolated during surgery. These cells have been extensively characterised as a model for human endothelial cells of the BBB [39]. Vials of cells at passage 28 were kindly supplied by Dr. P.O. Couraud (INSERM, Paris, France). Cells were grown and maintained in complete media at 37 °C and 5% CO_2_ and used between passages 29–35.

Confluent cells were treated with 0.02 ng/ml, 0.2 ng/ml, 2 ng/ml or 20 ng/ml recombinant human IL- 1β (Thermo Fisher) for 48 h and harvested for RNA extraction, followed by cDNA synthesis. In separate experiments, 1.2 µM clozapine (Abcam, Cambridge, UK), 26.6 nM haloperidol (Abcam) and 0.974 µM risperidone (Abcam) (based on typical therapeutic serum ranges) were used to treat the confluent culture for 48 h before harvesting the cells. This time point was chosen to avoid an immediate (acute) response to antipsychotic treatment. *ICAM1*, *ABCG2*, *CDH5*, *OCLN* and *IFITM* mRNA expression was measured by qPCR (supplementary information).

### sICAM1 protein in a living patient cohort

The recruitment and assessment of participants and analysis of their blood samples for this study was approved by the South Eastern Sydney and Illawara Area Health Services (HREC 07/259) and the University of New South Wales Human Research Ethics Committees (HREC 07121 and HREC 09187). Written informed consent was obtained from participants prior to participation. Plasma was collected from 78 chronically ill patients with schizophrenia or schizoaffective disorder and 73 healthy controls from the baseline time point of a previous study [40] and stored at −80 °C. Demographic information is available in Supplementary Table 3.

### sICAM protein assay

sICAM1 protein was measured with a Luminex Magpix-based assay (Luminex corporation, Austin, TX, USA) using a human magnetic luminex kit (R&D Systems, Minneapolis, MN, USA) according to manufacturer’s instruction (supplementary information). Briefly, plasma samples were thawed at 4 °C and centrifuged at 1400×*g* for 5 min. Supernatant was diluted at 1:8 in assay buffer and samples were assayed in duplicates. A 7-point standard curve with serial dilutions of 1:3 was used. Measurements were generated using the Millipore Analyst Software (Merck Millipore, Billerica, MA), using a 5 perimeter logistic standard curve corrected by blank background readings.

### Statistical analysis

Statistical analyses were performed using SPSS statistics (version 22; IBM, Armonk, NY, USA). DLPFC gene expressions were first analysed by diagnosis and then by inflammatory status. For diagnostic analysis, non-normally distributed data were transformed (log_10_: *ABCB1, ABCG2, ICAM1, OCLN, CDH5, IFITM, CD14, CD16* and *CD163* mRNAs). Group outliers defined as values greater than two standard deviations ± from the group mean were removed (0–4 per group) prior to analysis. Two-way ANOVAs were used to identify any effect of brain hemisphere or sex on mRNA transcripts. Demographic variables (age, PMI and RIN) were first tested to determine if they correlated with the expression of the gene of interest. If so, the demographic variable(s) were used as covariates in ANCOVA analyses of differential gene expression between diagnostic groups.

The postmortem cohort was previously defined as “high inflammation” schizophrenia (*n* = 14), “low inflammation” schizophrenia (*n* = 23) and “low inflammation” controls (*n* = 33) [14]. “High inflammation” controls were excluded from statistical analysis due to low sample size number (*n* = *4)*. The remaining three subgroups were not statistically significantly different in terms of age, sex distribution, brain hemisphere (left vs. right), PMI, or in terms of documented inflammatory symptoms in the week leading up to their death [14]. The three groups differed in tissue pH (*F*(2,67) = 7.563, *p* < 0.001) and therefore pH was not used as a covariate. For inflammatory subgroup analysis, non-normally distributed data were transformed (log_10_: *ABCB1, ABCG2, OCLN, CDH5, IFITM, CD14, CD16* and *CD163* mRNAs). ANCOVAs followed by Fisher’s least significant differences post-hoc tests were used to analyse differences due to diagnosis and inflammatory status. Welch’s ANOVA was used to confirm results for groups that did not meet the assumption of homogeneity of variance (Supplementary Table 4).

Cell culture experiments were analysed by one-way ANOVAs followed by Fisher’s least significant differences post-hoc tests. Frequencies of immunohistochemistry observations between diagnosis and by inflammatory groups were analysed by Chi square and Fisher’s exact test. In the living cohort of patients and healthy controls, sICAM1 was log transformed to achieve normality and an independent *t*-test was used to identify diagnostic group differences.

## Results

Correlations of brain endothelial cell and immune cell marker mRNAs with age, PMI, pH, RIN, antipsychotic dose, and illness duration and onset are presented in Supplementary Table 5. *IFITM* mRNA was significantly higher in the right hemisphere (*F*(1,65) = 6.39, *p* = 0.014). There was no significant effect of sex on any of the brain endothelial or immune cell transcripts (all *F*-values < 2.409, *p* > 0.25).

### Diagnostic differences in brain endothelial cell mRNAs

**Fig. 1:**
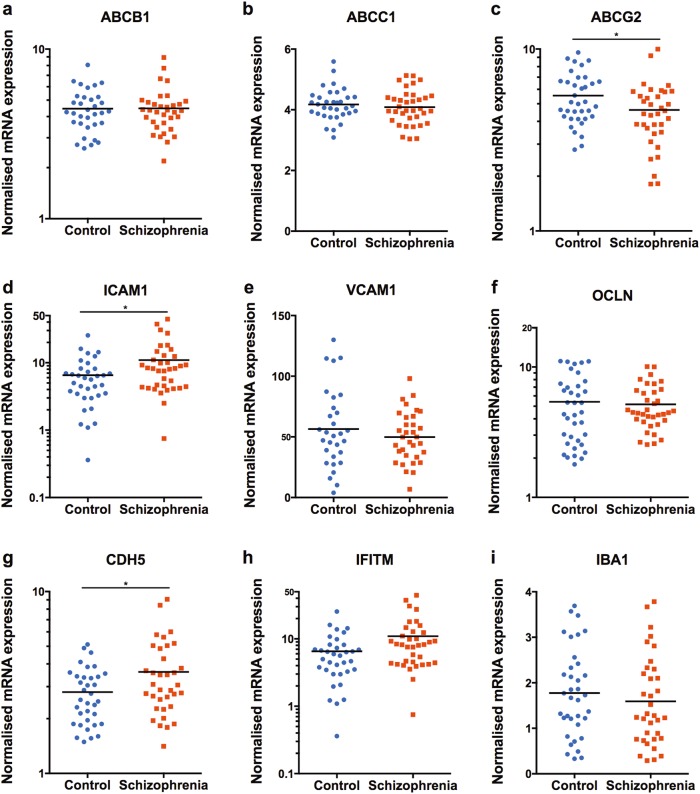
mRNA expression measured with qPCR in the DLPFC from schizophrenia and control brains of (**a**–**h**) brain endothelial cell genes and (**i**) *IBA1*. Note that *IFITM, ABCG2, ABCB1*, *ICAM1, CDH5* and *OCLN* are on log scales. *p < 0.05

The expression levels of several brain endothelial cell transcripts were statistically different between cases and controls in the DLPFC (Fig. 1a–h). We found a 68% increase in *ICAM1* mRNA in the schizophrenia group relative to controls, (ANCOVA: RIN *F*(1,68) = 6.295, *p* = 0.014) and a 29% increase in *CDH5* mRNA in schizophrenia subjects compared to controls (ANCOVA: RIN *F*(1,67) = 4.803, *p* = 0.032). In contrast, *ABCG2* mRNA was 17% lower in schizophrenia subjects compared to controls, (ANCOVA: age *F*(1,68) = 5.836, *p* = 0.018). There was no significant diagnostic difference between *ABCB1*, *ABCC1*, *IFITM*, *OCLN*, or *VCAM1* mRNA levels.

### More brain endothelial transcripts are altered in the subgroup of individuals with schizophrenia who have elevated cytokines

**Fig. 2:**
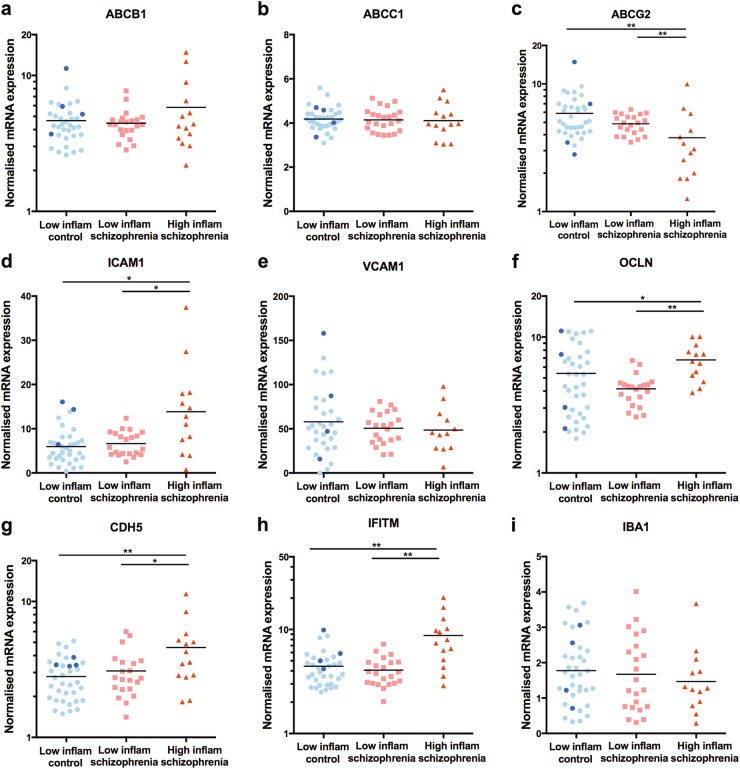
mRNA expression measured with qPCR in the DLPFC of (**a**–**h**) brain endothelial cell genes and (**i**) *IBA1* grouped according to low inflammation control, low inflammation schizophrenia and high inflammation schizophrenia subgroups based on cortical inflammatory marker expression. Data from high inflammation controls are shown in dark blue for illustrative purposes, but were excluded from statistical analysis due to the small group size. Note that *IFITM, ABCG2, ABCB1, CDH5* and *OCLN* are on log scales. *p < 0.05, **p < 0.01

Following analysis of subgroups based on elevated cytokine levels, we found additional transcripts that were differentially expressed in the DLPFC (Fig. 2a–h). *IFITM* expression was significantly increased in the “high inflammation” schizophrenia subgroup as compared to both the “low inflammation” schizophrenia subgroup (116% increase) and the “low inflammation” control subgroup (109% increase, ANCOVA: RIN, age (*F*(2,61) = 14.915, *p* < 0.001).

The “high inflammation” schizophrenia subgroup also had increased expression of *ICAM1* (ANCOVA: RIN *F*(2,63) = 12.128, *p* < 0.001) and decreased expression of *ABCG2* (ANCOVA: age *F*(2,62) = 10.935, *p* < 0.001) mRNA as compared to both the “low inflammation” schizophrenia subgroup (118% increase in *ICAM1* and 22% decrease in *ABCG2*) and “low inflammation” control subgroup (166% increase in *ICAM1* and 34% decrease in *ABCG2*). *CDH5* (ANCOVA: RIN *F*(2,62) = 5.783, *p* = 0.005) and *OCLN* (*F*(2,64) = 4.552, *p* = 0.014) mRNA expression levels in the “high inflammation schizophrenia” subgroup were significantly increased relative to the “low inflammation schizophrenia” subgroup (*CDH5*: 49%; *OCLN*: 63%) and the “low inflammation” control subgroup (*CDH5*: 69%; *OCLN*: 27%). We did not detect significant differences in levels of *ABCB1*, *ABCC1*, or *VCAM1* on the basis of the high and low inflammation subgroups.

### *ICAM1* expression is also elevated and associated with inflammatory status in the OFC

In the OFC, there was a 117% increase in *ICAM1* mRNA in people with schizophrenia compared to controls (ANCOVA: PMI *F*(1,67) = 9.838, *p* = 0.03). *ICAM1* expression in the OFC and DLPFC strongly and positively correlated across both patients and controls (*r* = 0.74, *p* < 0.001, df = 67). Following clustering by inflammatory markers, we confirmed that the “high inflammation” schizophrenia subgroup had greater *ICAM1* mRNA expression compared to the “low inflammation” schizophrenia subgroup and “low inflammation” control subgroup in the OFC (*F*(2,55) = 12.432, *p* < 0.001). This replicates our findings from the DLPFC in the OFC.

### Vascular and extravascular ICAM1 immunoreactivity

**Fig. 3:**
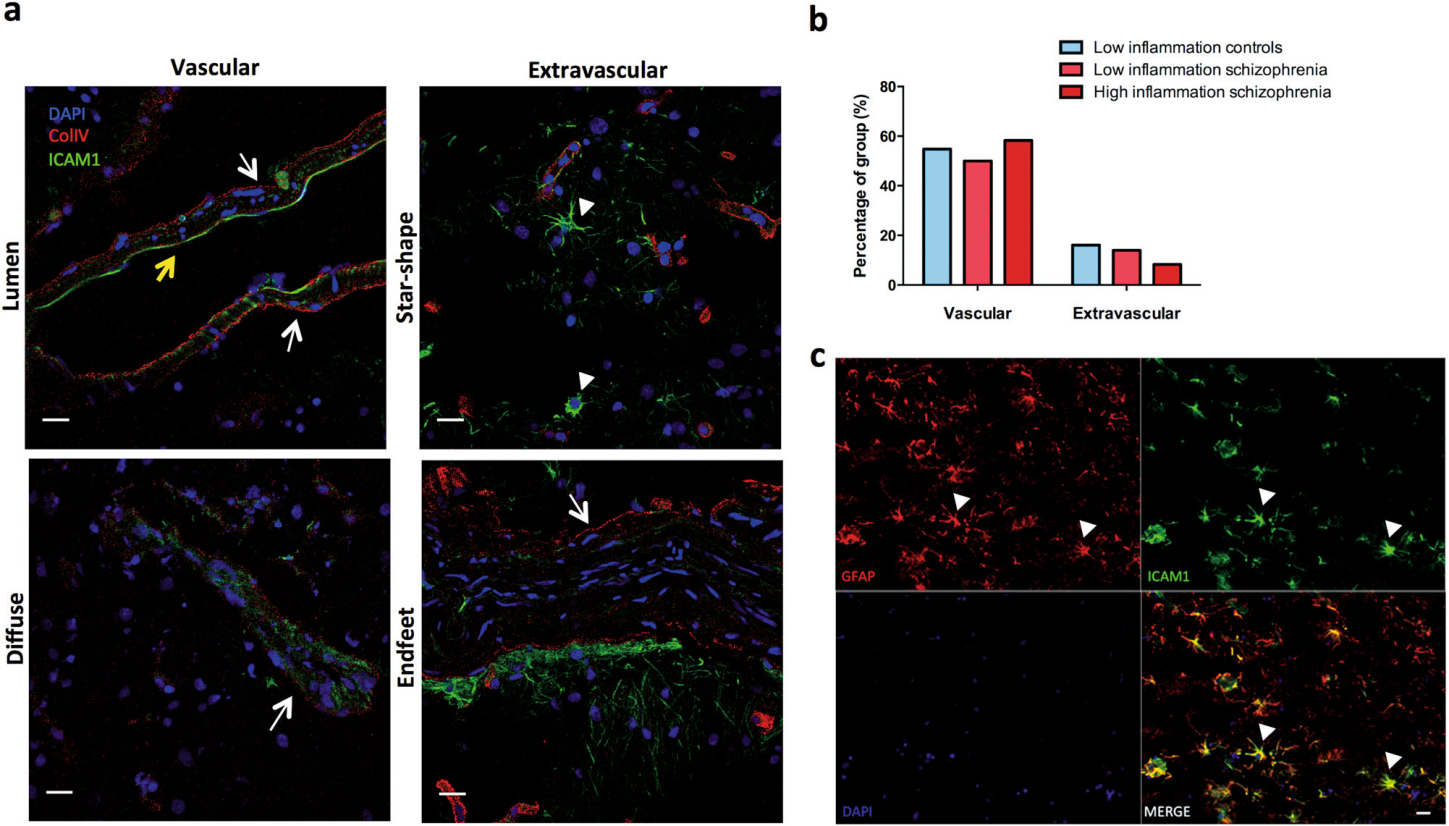
Distribution of ICAM1 immunostaining. **a** ICAM1 (green) and collagen IV (red) IHC staining was qualitatively assessed. Representative images illustrating ICAM1 distribution based on their localisation to vessels or astrocyte-like cells. Vascular ICAM1 was classified as having immunoreactivity situated in the lumen of the blood vessels surrounding endothelial cells (yellow arrow) or in a more diffuse pattern across the entire vessel (white arrows). Extravascular ICAM1 was classfied when ICAM1 immunoreactivity was observed in astrocyte-like cells (star-shaped, white arrowheads), in processes proximal to vessels or in processes that appear to be astrocytic endfeet attached to the vessels. We did not observe ICAM1 immunoreactivity near nuclei/cells indentifable as neuronal or microglia-like. Collagen-IV was used to label blood vessels as indicated by white arrows. ICAM1 attached to the lumen of the vessel is shown by the yellow arrow. White filled arrowheads indicate astrocyte-like cell. Scale bars are 20 µm. **b** There were no group differences in the frequency of vascular and extravascular ICAM1 immunoreactivity. **c** ICAM1 immunoreactivity (green in top right panel) with astrocyte like morphology (white arrowheads) co-localised with GFAP immunoreactivity (red intop left panel). DAPI stain in blue (bottom left panel) and merged image in bottom right panel. Scale bar is 20 µm

In human brain tissue, ICAM1 protein (green in Fig. 3a) co-localised with collagen IV to the brain vasculature (red in Fig. 3a). Additionally, there was extravascular ICAM1 immunoreactivity at cells with astrocyte-like morphology, cell processes and putative astrocytic endfeet found along blood vessels (Fig. 3a). GFAP double labelling with ICAM1 confirmed an overlap between ICAM1+ cells and some GFAP+ astrocytes (Fig. 3c).

Vascular associated ICAM1 immunoreactivity was more frequently observed compared to extravascular ICAM1 across all groups (Fig. 3b). The frequency of extravascular or vascular ICAM1 distribution did not differ between diagnoses or among “high inflammation” and “low inflammation” subgroups (Fig. 3b). However, extravascular ICAM1 was more prevalent in subjects older than 55 years (*χ*(1) = 4.987, *p* = 0.026), with only two cases under the age of 55 observed to have extravascular ICAM1 immunoreactivity. There were no age-dependent changes in vascular ICAM1, with vascular ICAM1 observed in 50% of cases under the age of 55 and 52% of cases over the age of 55.

### ICAM1 correlated with expression of an astrocyte but not a microglia marker

We assessed previously published GFAP mRNA data in this cohort [41] and found that *ICAM1* and *GFAP* mRNA transcripts correlated in DLPFC from controls (*r* = 0.40, *p* = 0.04) but not from people with schizophrenia (*r* = 0.26, *p* = 0.16). As ICAM1 has also been reported to be highly expressed in microglia [42], we measured the expression of the prototypical microglia marker, IBA1 and found *ICAM1* was not correlated with *IBA1* mRNA in controls (*r* = 0.20, *p* = 0.30) or schizophrenia (*r* = 0.13, *p* = 0.51). Further, there was no change in *IBA1* expression by diagnosis (*t*(65) = 0.711, *p* = 0.48; Fig. 1i) nor by inflammatory subgroups (*F*(2,64) = 0.401, *p* = 0.67; Fig. 2i).

### Effects of IL-1β and antipsychotic treatment on brain endothelial transcript expression

**Fig. 4:**
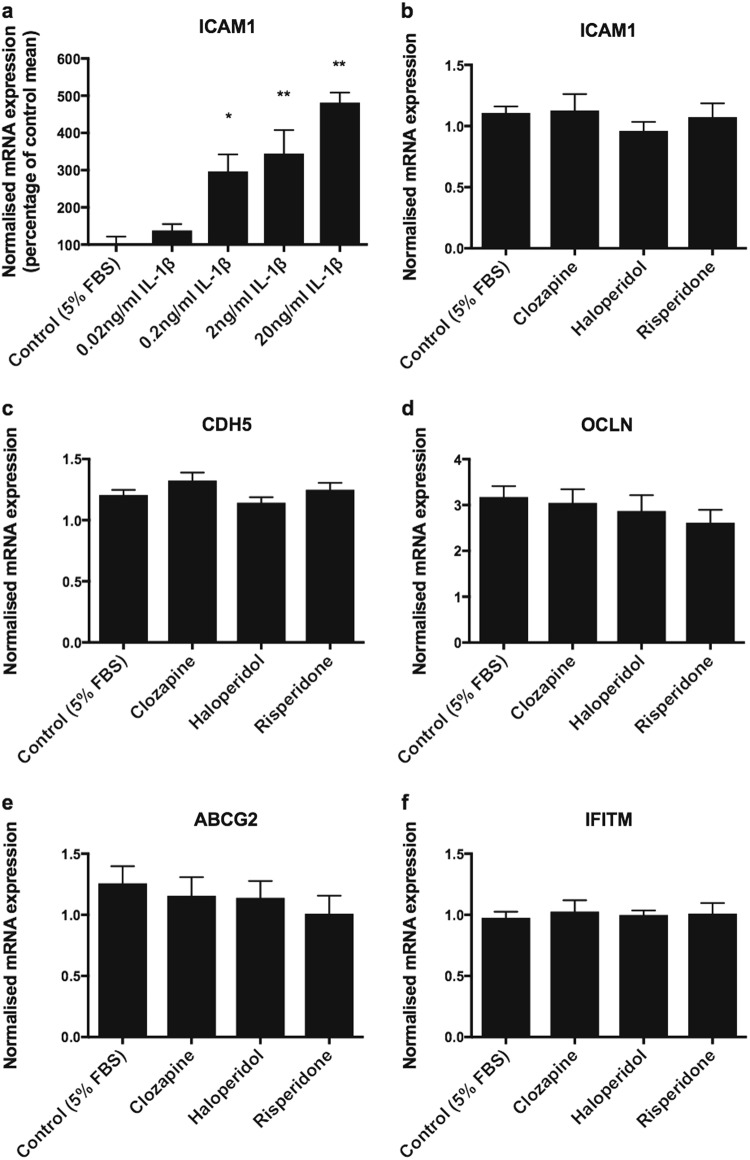
hCMEC/D3 cells treated with IL-1β and antipsychotics. **a** We found a dose-dependent increase in ICAM1 mRNA expression following 48 h incubations with IL-1β (*n* = 3 per treatment). *p < 0.05 compared to control (5% FBS), **p < 0.01 compared to control (5% FBS). **b–f** Antipsychotic treatment did not alter expression of brain endothelial cell genes in hCMEC/D3 cells (*n* = 8 per treatment). Data presented as mean ± SEM

In cultured human brain endothelial cells, *ICAM1* mRNA was up-regulated in a dose-dependent manner following 48 h of IL-1β incubation (*F*(4, 10) = 16.11, *p* < 0.001; Fig. 4a). In contrast, we found that when endothelial cells were incubated with therapeutic doses of various antipsychotics for the same time period, expressions of *ICAM1*, *CDH5*, *OCLN*, *ABCG2* or *IFITM* were unchanged (Fig. 4b–f).

### Immune cell marker mRNAs are elevated in high inflammation schizophrenia cases

**Fig. 5:**
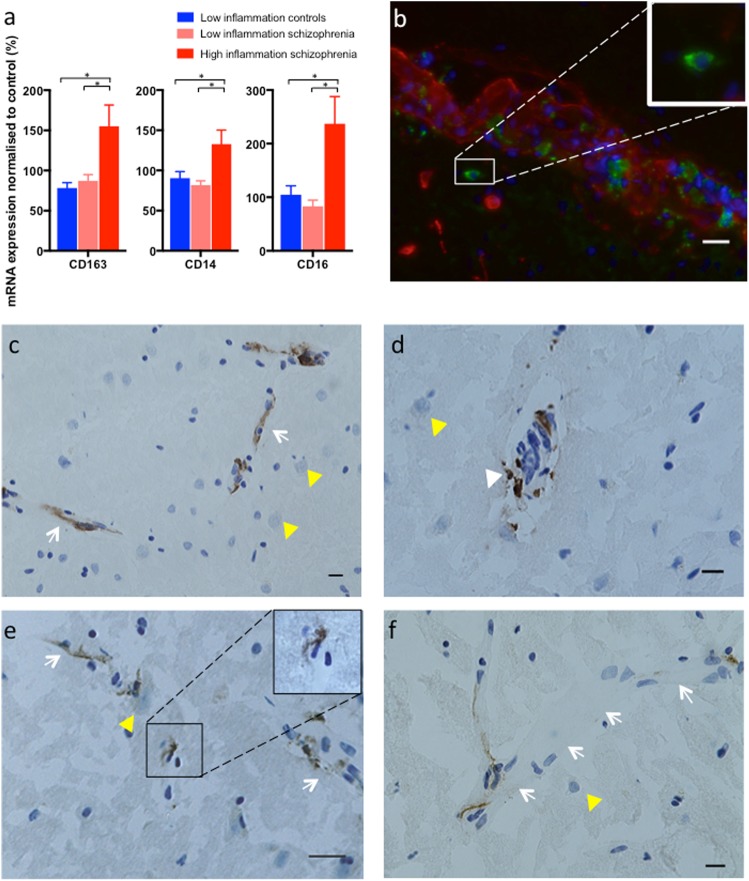
Markers of perivascular macrophages (CD163), activated monocytes (CD14) and monocytes/natural killer cells (CD16) are elevated in “high inflammation” schizophrenia. **a** Immune cell marker mRNAs were elevated in "high inflammation" schizophrenia compared to "low inflammation" schizophrenia and controls. Data presented as mean ± SEM, *p < 0.05. **b** Double-label immunofluorescence with CD163 (green) and collagen-IV (red) localised CD163+ cells inside the endothelium (red) and in the perivascular space (inset). **c** CD163+ cells were predominately found to be vascular (white arrows) with positive staining in all cases. **d** CD163+ cells could be found in the perivascular space and the parenchyma side of the blood vessel (white triangle). **e** A "high inflammation" schizophrenia case with a CD163+ macrophage found putatively in the parenchyma (inset) not associated with a blood vessel (white arrow). **f** Not all vessels have consistent CD163+ staining (white arrows demarcating a blood vessel). Neurons are marked by yellow triangles. Scale bars are 20 µm

Transcript levels of the three immune cell markers (*CD14, CD16* and *CD163*) were not significantly different between schizophrenia and controls (all *t* < 1.816, all *p* > 0. 074). However, stratifying by inflammatory subgroups revealed significant elevations in mRNAs of white blood cell markers in the “high inflammation” schizophrenia subgroup compared to the “low inflammation” schizophrenia and “low inflammation” control subgroups: *CD163* (ANCOVA: PMI *F*(2,61) = 6.288, *p* = 0.003), *CD16* (ANCOVA: PMI *F*(2,62) = 4.219, *p* = 0.019) and *CD14* (ANCOVA: RIN *F*(2,63) = 4.99, *p* = 0.01) (Fig. 5a).

### CD163+ perivascular macrophages in schizophrenia and control brains

Screening the gyrus rectus of the ventral medial prefrontal cortex for CD163 immunoreactive cells, we found these cells were predominately localised to blood vessels in every schizophrenia and control case examined (*n* = 76). Immunofluorescence double labelling confirmed CD163+ cells in both the vasculature and perivascular space (Fig. 5b). CD163+ cells were present in the perivascular space and the parenchymal side of the vasculature (Fig. 5c, d). We found CD163+ macrophages present in the parenchyma in close association with neurons and not associated with any blood vessels in 1 of 4 high inflammation controls (25%), 2 of 33 low inflammation controls (6%), 2 of 23 (9%) low inflammation schizophrenia cases and importantly in 6 of 14 (43%) high inflammation schizophrenia cases (*p* = 0.0096, Fisher’s exact test). Further, ICAM1 transcript levels were correlated with CD163 mRNA in the whole cohort (*r* = 0.42, *p* = 0.001) and also in the schizophrenia (*r* = 0.37, *p* = 0.036) and control (*r* = 0.40, *p* = 0.022) groups.

### sICAM1 is elevated in the plasma of living schizophrenia patients

**Fig. 6:**
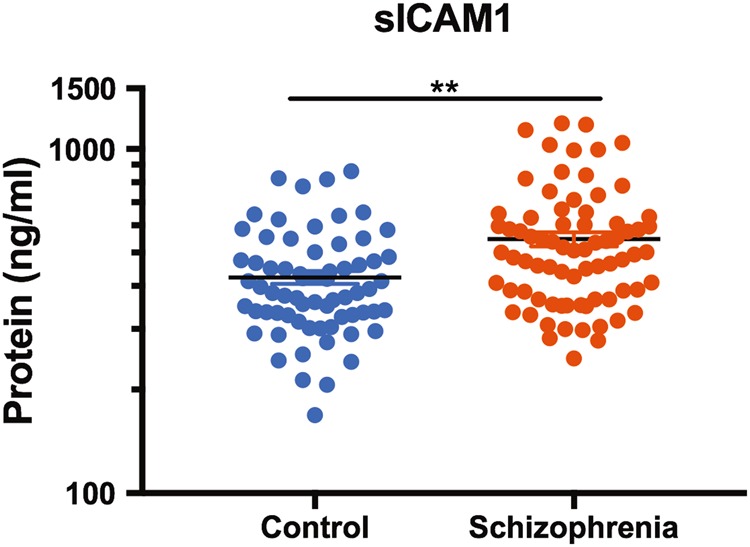
sICAM1 expression in plasma. sICAM1 protein was elevated in plasma from people with schizophrenia. Note that sICAM1 protein is on a log scale. **p < 0.01

sICAM1 was not correlated with age (*r* = 0.16, *p* = 0.07) nor was there a significant difference in sICAM1 between sexes (*F*(1,138) = 3, *p* = 0.09). There was a weak, trend level association between mean daily chlorpromazine equivalent dose and sICAM1 (rho = 0.22, *p* = 0. 054). sICAM1 was significantly upregulated in plasma of patients with schizophrenia relative to healthy controls (*t*(140) = 3.988, *p* = 0.0001; Fig. 6).

## Discussion

By measuring molecules enriched in brain endothelial cells involved in both the physical and functional properties of the BBB, we provide evidence supporting an altered BBB in people with schizophrenia. We find the greatest diagnostic change in *ICAM1* mRNA, which we confirmed to be localised to the lumen of human brain blood vessels and to be upregulated by cytokines but not antipsychotics in human brain endothelial cells in culture. We have also identified exaggerated transcriptional changes in *ABCG2*, *IFITM*, *ICAM1*, *OCLN, CDH5, CD163, CD14*, and *CD16* mRNAs in the “high inflammatory” subgroup of schizophrenia, demonstrating that some people with schizophrenia have greater molecular changes in the brain endothelial and white blood cell markers compared to others with schizophrenia.

While we focus on the inflammatory effects of cytokines related to the BBB, cytokines can have other neuromodulatory roles. Consistent with this, we have previously published that this “high inflammation” schizophrenia subgroup has greater neuropathology in the DLPFC, such as decreased brain derived neurotrophic factor mRNA, decreased interneuron markers, and evidence for astrogliosis, compared to the “low inflammation” schizophrenia subgroup [14, 41]. Inflammatory clustering was also conducted in the OFC of this cohort with similar overlap, showing neuroinflammation is not necessarily limited to the DLPFC and it is also associated with decreased cortical grey matter and superior frontal gyrus volume [15]. Living people with schizophrenia classified in the “high inflammation” biotype defined using blood-based inflammatory markers have reduced verbal fluency and reduced Broca’s area volume [13]. The mechanisms by which these established inflammatory genes contribute to these pathologies or vice versa remain unclear. A recent clinical trial using an IL-6 receptor antibody failed to demonstrate attenuated schizophrenia symptoms, potentially due to the lack of adequate antibody penetration into the brain [43]. However, there are many other reasons why this relatively small study was not able to demonstrate a beneficial effect, including low statistical power, dosing and time course issues, and the lack of stratifying patients based on inflammatory status prior to administering treatment, which may be necessary to achieve maximum benefit. Assessing neuroinflammation status may be a critical way to stratify people with schizophrenia who display a distinct or exaggerated neuropathology.

One of the most robust changes in this study was an increase in *ICAM1* mRNA in schizophrenia compared to controls. Further, the “high inflammation” schizophrenia subgroup had an even greater increase in *ICAM1* mRNA consistent with our own and other experimental results showing that IL-1β up-regulates *ICAM1* mRNA in cultured endothelial cells [44, 45]. ICAM1 has a role in the attachment of white blood cells to the luminal wall of blood vessels. We confirmed that brain endothelial cells are a cellular source of ICAM1 in the normal adult human brain and in the brains of people with schizophrenia. Infrequent failure to observe ICAM1 signal in the human cortex may be due to low sensitivity of the immunohistochemistry assay and because ICAM1 is normally expressed at low levels and is actively cleaved. ICAM1 also co-localises with astrocytes, an observation more frequent in brains from older individuals. Previous studies found increased extravascular ICAM1 with aging [46, 47], and that *GFAP* mRNA and protein also increase with age [41, 48, 49]. While ICAM1 can also be expressed in microglia, we did not find ICAM1 to be localised to microglial cells based on morphology. Further, there was no correlation between *ICAM1* and *IBA1* mRNA. *IBA1* mRNA was not changed in our cohort and postmortem studies quantifying microglia density using IBA1 in schizophrenia find no differences [50, 51], though increased microglia density have been observed when using other markers [14]. Our results suggest that beyond endothelial cells, astrocytes and not microglia are the main cellular source of ICAM1 in brain tissue.

Conflicting findings exist as to whether or not peripheral sICAM1 levels are increased, decreased or not changed in schizophrenia relative to controls. Our finding of elevated plasma sICAM1 in chronically ill people with schizophrenia supports studies showing elevated peripheral sICAM1 levels in patients receiving antipsychotic medication [31], and patients with schizophrenia who have a putative disruption of the BBB [28]. While most previous studies have investigated serum, a study in plasma also finds elevated sICAM1 in chronically ill schizophrenia patients [32], consistent with our measures in plasma from chronically ill patients. However, others have reported decreased serum sICAM1 levels in medicated and un-medicated patients with schizophrenia compared to controls [28, 29]. There is also evidence that sICAM1 may vary according to clinical features; for example, lower sICAM1 is linked to better treatment outcomes [30] supporting the hypothesis that higher sICAM1 may be deleterious to brain function.

Reduced sICAM1 was also found in schizophrenia patients after switching to second generation antipsychotics [52]. In our study, we found that peripheral sICAM levels are positively correlated with antipsychotic dose levels at a borderline level of significance. Thus, a higher dose of antipsychotics may elicit an increase in sICAM1, or conversely, elevated sICAM1 (putatively indicative of greater leucocyte extravasation into brain tissue) may require increased doses of antipsychotics. Our experimental results in cultured cells derived from human brain endothelium suggest that antipsychotic drugs may not directly increase ICAM1 expression, but further experiments are needed to determine how they may influence the production and cleavage of the soluble form of ICAM1 in these cells. In summary, the results from studies of sICAM1 in schizophrenia are divergent and may reflect a number of factors. The influence of antipsychotic treatment is unclear and a more systematic approach to research on this topic is required. Our findings support that schizophrenia may have an underlying inflammatory component, but not everyone with schizophrenia is in a high inflammatory state or has clear changes in their brain vasculature or in circulating inflammatory factors like sICAM1.

Membrane bound ICAM1 interacts with lymphocyte function-associated antigen-1 (LFA1) and macrophage associated antigen-1 (MAC1) receptors expressed on leucocytes to promote immune cell infiltration during tissue inflammation [53]. Elevated sICAM1 in the plasma of people with schizophrenia may reflect enhanced cleavage of membrane bound ICAM1 on the endothelium following recruitment of immune cells into the tissue. sICAM1 also interacts with LFA1 and MAC1, thereby interfering with the ability of leucocytes to bind to membrane bound ICAM1. As such, sICAM1 may contribute to the increased circulation of activated monocytes reported in schizophrenia [54, 55] and promote release of pro-inflammatory cytokines from immune cells, further contributing to the inflammatory state.

CD3+ T lymphocytes and CD20+ B lymphocytes have been observed in the hippocampus in a small number of schizophrenia brains [33] and in a broader investigation in the hippocampus, frontal cortex, thalamus, medial temporal lobe and cingulate gyrus in schizophrenia [34]. Inflammation related genes including *S100A8*, *S100A9* and *CHI3L1* are elevated in the hippocampus in schizophrenia and perivascular macrophages (CD163+) have been identified in the lumen of blood vessels and surrounding the endothelial cells in at least one schizophrenia hippocampus [21].

In our study, transcript levels of *CD16*, a marker of natural killer cells and activated macrophages/monocytes, *CD163* a marker of perivascular macrophages and *CD14*, a marker of monocytes were all elevated in schizophrenia cases with high inflammation. CD163+ macrophages were detected in the brain vasculature in all cases irrespective of diagnosis and inflammation. Our study now confirms that monocytes can also be found in the prefrontal cortex of individuals with schizophrenia and for the first time, identifies brain tissue macrophages proximal to neurons in over 40% of individuals with schizophrenia who are in a high inflammatory state.

Our anatomical scope of analysis is restricted as we used 14 µm tissue sections and sampled only a small part (rostro-caudally) of the gyrus rectus microscopically and thus, we cannot comment on the total number of CD163+ cells in the brains of people with schizophrenia. Determining the actual number of cells would require counting using stereological principles, having an entire known volume of the brain area of interest available and systematic sampling of multiple tissue sections. This would require resources (both time and space) that are not currently tractable with the limited amount of human brain tissue typically available for study. We found CD163+ macrophages in the brain parenchyma away from any obvious blood vessels, suggesting that they are capable of infiltrating brain tissue and interacting directly with neurons. Breakdown of the BBB is not a prerequisite for leucocyte infiltration and bone marrow-derived monocytes can access the (rodent) brain without changes to BBB permeability [56]. Therefore, our findings do not necessarily suggest a breakdown or leakiness of the BBB, but rather more potential for circulating immune cells to adhere to the blood vessel endothelium. The presence of immune cells in brain tissue can produce inflammatory factors to further drive the inflammatory cascade by signalling to microglia, astrocytes and back to the endothelium.

Tight junction proteins such as *CDH5* and *OCLN* that contribute to BBB integrity were found to be elevated in relation to inflammation in postmortem tissue in schizophrenia. While this may suggest a tightening of the BBB, it could also be a compensatory response to prevent excessive entry of leucocytes into the perivascular space under inflammatory conditions, or greater utilisation and turnover of these molecules leading to the need for higher synthesis of tight junction proteins in inflammatory states. In addition to cytokine measures, C-reactive protein is a peripheral inflammation marker that could be used to determine inflammatory status and it may be informative for future studies to collect blood for peripheral measures of inflammation along with the brain at time of death. As BBB integrity can be indirectly measured in vivo using novel MRI technology [57], we suggest that MRI could be used with blood cytokine and C-reactive protein measures to determine whether changes in BBB integrity or function and elevated inflammatory status co-exist in living individuals with schizophrenia.

A limitation of our study is that all individuals with schizophrenia were prescribed antipsychotics and were chronically ill. Chlorpromazine equivalent dose positively correlated with *ICAM1, IFITM* and *CDH5* mRNA levels in the postmortem sample, and there was a weak, positive correlation of mean daily chlorpromazine equivalent dose with sICAM1 at a trend level in the living sample. However, exposing cultured endothelial cells to three different antipsychotics did not alter the expression of any genes of interest. Furthermore, all patients were receiving antipsychotics and several mRNAs including *ICAM1, IFITM* and *CDH5* differed significantly between the two inflammatory patient groups. We suggest that antipsychotics alone may not be a primary factor in regulating gene expression of brain endothelial cell transcripts.

Another limitation of our study is the use of tissue homogenates for mRNA experiments, which does not provide cell-specific data. Consulting the Human Protein Atlas indicates that the expressions of brain endothelial markers of interest are predominately found in endothelial cells of the human brain [58]. A previous study using laser microdissection to examine the brain microvasculature finds a down-regulation in inflammation related genes in schizophrenia compared to controls [17] and is in apparent contradiction to our study utilising brain homogenates. However, the status of cytokine or inflammatory mRNAs within the blood endothelial cells was not provided in this previous study and it may be that cytokines are also or alternatively elevated in neuronal or glial cells.

The stress from a lifetime of mental illness may contribute to inflammation in the brain in our chronically ill cohort. While peripheral cortisol levels can be elevated in schizophrenia [59], glucocorticoid receptor mRNA levels are also dysregulated in the brains of people with schizophrenia [60, 61]. When using changes in cortical glucocorticoid and immune pathways to stratify subgroups, we find an overlap in the population of people with schizophrenia in an elevated inflammatory state and in the sample of people with schizophrenia in an elevated stress state [10]. Further research is required to disentangle the reciprocal role of inflammation and stress in the neuropathology of schizophrenia.

In contrast to previous studies [4, 16, 21, 62], we did not detect any diagnostic differences in *IFITM* mRNA expression in people with schizophrenia compared to controls. This is possibly because our pan-probe measured *IFITM1*, *IFITM2*, and *IFITM3* transcripts. In contrast, using qPCR Siegel et al [62]. found an increase in *IFITM1* expression and with a pan-probe in *IFITM2* and *IFITM3* expression in schizophrenia. This group also found that IFITM2 mRNA is predominantly expressed in the brain vasculature by in situ hybridisation. *IFITM* expression can be induced by inflammatory cytokines, IL-1β [63] and IL-6 [64], the same cytokines used to define inflammatory status in our cohort [14]. In support of these findings, we found a significant increase in *IFITM* expression in the “high inflammation” schizophrenia subgroup compared to the “low inflammation” schizophrenia subgroup.

We are the first to report changes in efflux transporters mRNAs in the brains of people with schizophrenia compared to controls, with greatest decreases in cases with “high inflammation”. The exacerbated decrease in *ABCG2* in the “high inflammation” schizophrenia subgroup is consistent with evidence showing that treatment with IL-1β, IL-6 and TNFα reduces both mRNA and protein expression of endothelial ABCG2 in vitro [65]. We did not detect a change in either *ABCB1* or *ABCC1* expression in people with schizophrenia nor in the context of high inflammation, which is in keeping with the fact that *ABCB1* expression is only slightly reduced by IL-6 and increased by TNFα [65]. ABCG2 interacts with substrates in the cell cytoplasm [66] allowing substrates not initially filtered by ABCB1 to be removed. Lower levels of ABCG2 especially in the context of elevated inflammation may result in less capacity to filter out and remove unwanted molecules in the prefrontal cortex for some people with schizophrenia. This dysfunction may delay the brain’s capacity to resolve inflammation and/or could exacerbate existing damage.

In conclusion, we are the first to report molecular alterations in brain endothelial cells and in monocyte/macrophage markers in the PFC from people with schizophrenia that are exaggerated when other signs of inflammation are present. Elevated *ICAM1* does not necessarily prove that the BBB is more leaky in individuals with schizophrenia, but instead supports the hypothesis that luminal walls of blood vessels have more potential for increased capture of immune cells in the disease state. Further, we have identified CD163+ macrophages in the parenchyma of some schizophrenia brains. Multiple studies have shown substantial proportions of patients with schizophrenia have peripheral immune system changes and our study supports that these are not necessarily independent of neural changes, but rather that the two are inter-related. Further characterisation of these individuals utilising measurements in both brain and blood will contribute to our understanding of how elevated neuroinflammation contributes to the heterogeneity and pathophysiology of schizophrenia.

## Electronic supplementary material

Supplementary methods

Supplementary figure 1

Supplementary table 1

Supplementary table 2

Supplementary table 3

Supplementary table 4

Supplementary table 5

## References

[1] Erbagcica AB, Herken H, Köylüoglu O, Yilmaz N, Tarakçioglu M (2001). Serum IL-1β, sIL-2R, IL-6, IL-8 and TNF-α in schizophrenic patients, relation with symptomatology and responsiveness to risperidone treatment. Mediat Inflamm.

[2] Na KS, Jung HY, Kim YK (2014). The role of pro-inflammatory cytokines in the neuroinflammation and neurogenesis of schizophrenia. Prog Neuropsychopharmacol Biol Psychiatry.

[3] Kunz M, Cereser KM, Goi PD, Fries GR, Teixeira AL, Fernandes BS (2011). Serum levels of IL-6, IL-10 and TNF-alpha in patients with bipolar disorder and schizophrenia: differences in pro- and anti-inflammatory balance. Rev Bras Psiquiatr.

[4] Saetre P, Emilsson L, Axelsson E, Kreuger J, Lindholm E, Jazin E (2007). Inflammation-related genes up-regulated in schizophrenia brains. BMC Psychiatry.

[5] Schizophrenia Psychiatric Genome-Wide Association Study C. (2011). Genome-wide association study identifies five new schizophrenia loci. Nat Genet.

[6] Nawa H, Takei N (2006). Recent progress in animal modeling of immune inflammatory processes in schizophrenia: implication of specific cytokines. Neurosci Res.

[7] Trepanier MO, Hopperton KE, Mizrahi R, Mechawar N, Bazinet RP (2016). Postmortem evidence of cerebral inflammation in schizophrenia: a systematic review. Mol Psychiatry.

[8] Brown AS, Derkits EJ (2010). Prenatal infection and schizophrenia: a review of epidemiologic and translational studies. Am J Psychiatry.

[9] Brown AS, Hooton J, Schaefer CA, Zhang H, Petkova E, Babulas V (2004). Elevated maternal interleukin-8 levels and risk of schizophrenia in adult offspring. Am J Psychiatry.

[10] Fillman SG, Sinclair D, Fung SJ, Webster MJ, Shannon Weickert C (2014). Markers of inflammation and stress distinguish subsets of individuals with schizophrenia and bipolar disorder. Transl Psychiatry.

[11] Horvath S, Mirnics K (2014). Immune system disturbances in schizophrenia. Biol Psychiatry.

[12] Bechter K, Reiber H, Herzog S, Fuchs D, Tumani H, Maxeiner HG (2010). Cerebrospinal fluid analysis in affective and schizophrenic spectrum disorders: identification of subgroups with immune responses and blood-CSF barrier dysfunction. J Psychiatr Res.

[13] Fillman SG, Weickert TW, Lenroot RK, Catts SV, Bruggemann JM, Catts VS (2016). Elevated peripheral cytokines characterize a subgroup of people with schizophrenia displaying poor verbal fluency and reduced Broca's area volume. Mol Psychiatry.

[14] Fillman SG, Cloonan N, Catts VS, Miller LC, Wong J, McCrossin T (2013). Increased inflammatory markers identified in the dorsolateral prefrontal cortex of individuals with schizophrenia. Mol Psychiatry.

[15] Zhang Y, Catts VS, Sheedy D, McCrossin T, Kril JJ, Shannon Weickert C (2016). Cortical grey matter volume reduction in people with schizophrenia is associated with neuro-inflammation. Transl Psychiatry.

[16] Volk DW, Chitrapu A, Edelson JR, Roman KM, Moroco AE, Lewis DA (2015). Molecular mechanisms and timing of cortical immune activation in schizophrenia. Am J Psychiatry.

[17] Harris LW, Wayland M, Lan M, Ryan M, Giger T, Lockstone H (2008). The cerebral microvasculature in schizophrenia: a laser capture microdissection study. PLoS One.

[18] Najjar S, Pearlman DM, Alper K, Najjar A, Devinsky O (2013). Neuroinflammation and psychiatric illness. J Neuroinflamm.

[19] Hanson DR, Gottesman II (2005). Theories of schizophrenia: a genetic-inflammatory-vascular synthesis. BMC Med Genet.

[20] Uranova NA, Zimina IS, Vikhreva OV, Krukov NO, Rachmanova VI, Orlovskaya DD (2010). Ultrastructural damage of capillaries in the neocortex in schizophrenia. World J Biol Psychiatry.

[21] Hwang Y, Kim J, Shin JY, Kim JI, Seo JS, Webster MJ (2013). Gene expression profiling by mRNA sequencing reveals increased expression of immune/inflammation-related genes in the hippocampus of individuals with schizophrenia. Transl Psychiatry.

[22] Dufour A, Corsini E, Gelati M, Ciusani E, Zaffaroni M, Giombini S (1998). Modulation of ICAM-1, VCAM-1 and HLA-DR by cytokines and steroids on HUVECs and human brain endothelial cells. J Neurol Sci.

[23] Min JK, Kim YM, Kim SW, Kwon MC, Kong YY, Hwang IK (2005). TNF-related activation-induced cytokine enhances leukocyte adhesiveness: induction of ICAM-1 and VCAM-1 via TNF receptor-associated factor and protein kinase C-dependent NF-kappaB activation in endothelial cells. J Immunol.

[24] Labus J, Hackel S, Lucka L, Danker K (2014). Interleukin-1beta induces an inflammatory response and the breakdown of the endothelial cell layer in an improved human THBMEC-based in vitro blood-brain barrier model. J Neurosci Methods.

[25] Shaftel SS, Carlson TJ, Olschowka JA, Kyrkanides S, Matousek SB, O'Banion MK (2007). Chronic interleukin-1beta expression in mouse brain leads to leukocyte infiltration and neutrophil-independent blood brain barrier permeability without overt neurodegeneration. J Neurosci.

[26] Engelhardt B, Ransohoff RM (2012). Capture, crawl, cross: the T cell code to breach the blood-brain barriers. Trends Immunol.

[27] Engelhardt B, Ransohoff RM (2005). The ins and outs of T-lymphocyte trafficking to the CNS: anatomical sites and molecular mechanisms. Trends Immunol.

[28] Schwarz MJ, Riedel M, Ackenheil M, Muller N (2000). Decreased levels of soluble intercellular adhesion molecule-1 (sICAM-1) in unmedicated and medicated schizophrenic patients. Biol Psychiatry.

[29] Kronig H, Riedel M, Schwarz MJ, Strassnig M, Moller HJ, Ackenheil M (2005). ICAM G241A polymorphism and soluble ICAM-1 serum levels: evidence for an active immune process in schizophrenia. Neuroimmunomodulation.

[30] Stefanovic MP, Petronijevic N, Dunjic-Kostic B, Velimirovic M, Nikolic T, Jurisic V (2016). Role of sICAM-1 and sVCAM-1 as biomarkers in early and late stages of schizophrenia. J Psychiatr Res.

[31] Kavzoglu SO, Hariri AG (2016). Intracellular Adhesion Molecule (ICAM-1), Vascular Cell Adhesion Molecule (VCAM-1) and E-Selectin Levels in First Episode Schizophrenic Patients. Klin Psikofarmakol Bülteni-Bull Clin Psychopharmacol.

[32] Nguyen TT, Dev SI, Chen G, Liou SC, Martin AS, Irwin MR et al. Abnormal levels of vascular endothelial biomarkers in schizophrenia. Eur Arch Psychiatry Clin Neurosci. 2017. 10.1007/s00406-017-0842-6 10.1007/s00406-017-0842-6 PMC8023592 28942562

[33] Busse S, Busse M, Schiltz K, Bielau H, Gos T, Brisch R (2012). Different distribution patterns of lymphocytes and microglia in the hippocampus of patients with residual versus paranoid schizophrenia: further evidence for disease course-related immune alterations?. Brain Behav Immun.

[34] Bogerts B, Winopal D, Schwarz S, Schlaaff K, Dobrowolny H, Mawrin C (2017). Evidence of neuroinflammation in subgroups of schizophrenia and mood disorder patients: a semiquantitative postmortem study of CD3 and CD20 immunoreactive lymphocytes in several brain regions. Neurol Psychiatry Brain Res.

[35] Lampugnani MG, Resnati M, Raiteri M, Pigott R, Pisacane A, Houen G (1992). A novel endothelial-specific membrane protein is a marker of cell-cell contacts. J Cell Biol.

[36] Loscher W, Potschka H (2005). Blood-brain barrier active efflux transporters: ATP-binding cassette gene family. NeuroRx.

[37] Hammond ME, Lapointe GR, Feucht PH, Hilt S, Gallegos CA, Gordon CA (1995). IL-8 induces neutrophil chemotaxis predominantly via type I IL-8 receptors. J Immunol.

[38] Weickert CS, Sheedy D, Rothmond DA, Dedova I, Fung S, Garrick T (2010). Selection of reference gene expression in a schizophrenia brain cohort. Aust N Z J Psychiatry.

[39] Weksler B, Romero IA, Couraud PO (2013). The hCMEC/D3 cell line as a model of the human blood brain barrier. Fluids Barriers CNS.

[40] Weickert TW, Weinberg D, Lenroot R, Catts SV, Wells R, Vercammen A (2015). Adjunctive raloxifene treatment improves attention and memory in men and women with schizophrenia. Mol Psychiatry.

[41] Catts VS, Wong J, Fillman SG, Fung SJ, Shannon Weickert C (2014). Increased expression of astrocyte markers in schizophrenia: Association with neuroinflammation. Aust N Z J Psychiatry.

[42] Zielasek J, Archelos JJ, Toyka KV, Hartung HP (1993). Expression of intercellular adhesion molecule-1 on rat microglial cells. Neurosci Lett.

[43] Girgis RR, Ciarleglio A, Choo T, Haynes G, Bathon JM, Cremers S, et al. A randomized, double-blind, placebo-controlled clinical trial of tocilizumab, an interleukin-6 receptor antibody, for residual symptoms in schizophrenia. Neuropsychopharmacology. 2018;43:1317–1323. 10.1038/npp.2017.258 PMC5916349 29090685

[44] Myers CL, Wertheimer SJ, Schembri-King J, Parks T, Wallace RW (1992). Induction of ICAM-1 by TNF-alpha, IL-1 beta, and LPS in human endothelial cells after downregulation of PKC. Am J Physiol.

[45] Wang X, Feuerstein GZ, Gu JL, Lysko PG, Yue TL (1995). Interleukin-1 beta induces expression of adhesion molecules in human vascular smooth muscle cells and enhances adhesion of leukocytes to smooth muscle cells. Atherosclerosis.

[46] Miguel-Hidalgo JJ, Nithuairisg S, Stockmeier C, Rajkowska G (2007). Distribution of ICAM-1 immunoreactivity during aging in the human orbitofrontal cortex. Brain Behav Immun.

[47] Miguel-Hidalgo JJ, Overholser JC, Jurjus GJ, Meltzer HY, Dieter L, Konick L (2011). Vascular and extravascular immunoreactivity for intercellular adhesion molecule 1 in the orbitofrontal cortex of subjects with major depression: age-dependent changes. J Affect Disord.

[48] David JP, Ghozali F, FalletBianco C, Wattez A, Delaine S, Boniface B (1997). Glial reaction in the hippocampal formation is highly correlated with aging in human brain. Neurosci Lett.

[49] Hansen LA, Armstrong DM, Terry RD (1987). An immunohistochemical quantification of fibrous astrocytes in the aging human cerebral cortex. Neurobiol Aging.

[50] Connor CM, Guo Y, Akbarian S (2009). Cingulate white matter neurons in schizophrenia and bipolar disorder. Biol Psychiatry.

[51] Hercher C, Chopra V, Beasley CL (2014). Evidence for morphological alterations in prefrontal white matter glia in schizophrenia and bipolar disorder. J Psychiatry Neurosci.

[52] Meyer U, Schwarz MJ, Müller N (2011). Inflammatory processes in schizophrenia: a promising neuroimmunological target for the treatment of negative/cognitive symptoms and beyond. Pharmacol Ther.

[53] Hermand P, Huet M, Callebaut I, Gane P, Ihanus E, Gahmberg CG (2000). Binding sites of leukocyte beta 2 integrins (LFA-1, Mac-1) on the human ICAM-4/LW blood group protein. J Biol Chem.

[54] Zorrilla EP, Cannon TD, Gur RE, Kessler J (1996). Leukocytes and organ-nonspecific autoantibodies in schizophrenics and their siblings: Markers of vulnerability or disease?. Biol Psychiatry.

[55] Miller BJ, Gassama B, Sebastian D, Buckley P, Mellor A (2013). Meta-analysis of lymphocytes in schizophrenia: clinical status and antipsychotic effects. Biol Psychiatry.

[56] Wohleb ES, Powell ND, Godbout JP, Sheridan JF (2013). Stress-induced recruitment of bone marrow-derived monocytes to the brain promotes anxiety-like behavior. J Neurosci.

[57] Montagne A, Barnes SR, Sweeney MD, Halliday MR, Sagare AP, Zhao Z (2015). Blood-brain barrier breakdown in the aging human hippocampus. Neuron.

[58] Uhlen M, Fagerberg L, Hallstrom BM, Lindskog C, Oksvold P, Mardinoglu A (2015). Proteomics. Tissue-based map of the human proteome. Science.

[59] Bradley AJ, Dinan TG (2010). A systematic review of hypothalamic-pituitary-adrenal axis function in schizophrenia: implications for mortality. J Psychopharmacol.

[60] Sinclair D, Tsai SY, Woon HG, Weickert CS (2011). Abnormal glucocorticoid receptor mRNA and protein isoform expression in the prefrontal cortex in psychiatric illness. Neuropsychopharmacology.

[61] Sinclair D, Fillman SG, Webster MJ, Weickert CS (2013). Dysregulation of glucocorticoid receptor co-factors FKBP5, BAG1 and PTGES3 in prefrontal cortex in psychotic illness. Sci Rep.

[62] Siegel BI, Sengupta EJ, Edelson JR, Lewis DA, Volk DW (2014). Elevated viral restriction factor levels in cortical blood vessels in schizophrenia. Biol Psychiatry.

[63] Ranjbar S, Haridas V, Jasenosky LD, Falvo JV, Goldfeld AE (2015). A role for IFITM proteins in restriction of Mycobacterium tuberculosis Infection. Cell Rep.

[64] Diamond MS, Farzan M (2013). The broad-spectrum antiviral functions of IFIT and IFITM proteins. Nat Rev Immunol.

[65] Poller B, Drewe J, Krahenbuhl S, Huwyler J, Gutmann H (2010). Regulation of BCRP (ABCG2) and P-glycoprotein (ABCB1) by cytokines in a model of the human blood-brain barrier. Cell Mol Neurobiol.

[66] Mikitsh JL, Chacko AM (2014). Pathways for small molecule delivery to the central nervous system across the blood-brain barrier. Perspect Med Chem.

