# Supplementary material for: Increased macrophages and changed brain endothelial cell gene expression in the frontal cortex of people with schizophrenia displaying inflammation
Source: Mol Psychiatry. 2018 Sep 13;25(4):761–75. doi: 10.1038/s41380-018-0235-x (PMC7156343; doi:10.1038/s41380-018-0235-x)
Supplement: Supplementary file 1 — Supplementary methods [file 41380_2018_235_MOESM1_ESM.docx]

**Supplementary methods**

**Endothelial cell culture**

Complete media was made up in endothelial basal medium-2 (Lonza, Mt Waverly, VIC, AUS) supplemented with 5% fetal bovine serum (Thermo Fisher), 1.4 µM hydrocortisone (Sigma-Aldrich), 5 µg/ml ascorbic acid (Sigma-Aldrich), 1% chemically defined lipid concentration (Thermo Fisher), 10 mM HEPES (Thermo Fisher), 1 ng/ml bFGF (Sigma-Aldrich) and 0.1 mg/ml penicillin/streptomycin (Thermo Fisher).

**Treatment of hCMEC/D3 cells with IL-1β**

Cells were seeded at 0.7 x 10^6^ cells/ml on 300 µg/ml Cultrex Rat Collagen I (Trevigen, Gaithersburg, MD, USA) coated T25 flasks (Corning, Corning, NY, USA) and grown for 48 hours in complete media. Then, cells were treated (replicates and repeated for final n = 8) with 0.02 ng/ml, 0.2 ng/ml, 2 ng/ml or 20 ng/ml recombinant human IL-1β (Thermo Fisher) or complete media for an additional 48 hours before cells were washed with warm Dulbecco’s PBS (Thermo Fisher) and harvested with TRIzol for RNA extraction.

RNA quality was measured using an Agilent 2100 bioanalyzer (RINs ranged from 9.6-10). Complementary DNA was generated using a SuperScript First-Strand Synthesis kit (Thermo Fisher) as described previously^31^ and mRNA was measured using qPCR (triplicate measures per sample). The averaged raw data was normalized to the geomean of four housekeeper control mRNAs (β-actin, peptidylprolyl isomerase A, ubiquitin C, and β2 microglobulin).

**Treatment of hCMEC/D3 cells with antipsychotics**

Common antipsychotic (clozapine, haloperidol, and risperidone) concentrations were chosen to reflect the typical therapeutic serum range.^33-36^ Stock solutions were made up in 100% ethanol and diluted with complete media to final concentrations of 1.2 µM clozapine (Abcam, Cambridge, UK), 26.6 nM haloperidol (Abcam) and 0.974 µM risperidone (Abcam). Prior to treatment, cells were seeded at 0.7 x 10^6^ cells/ml on 300 µg/ml Cultrex Rat Collagen I (Trevigen) coated T25 flasks (Corning) and grown for 48 hours. Media was replaced with antipsychotic-supplemented media for an additional 48 hours before harvesting and preparation for qPCR as described above. This time point was chosen to avoid an immediate (acute) response to antipsychotic treatment. mRNA expression was measured for *ICAM1*, *ABCG2*, *CDH5*, *OCLN* and *IFITM*.

**Magnetic luminex assay**

Samples were incubated with a human magnetic premixed microparticle cocktail in each well for 2 hours at room temperature with shaking. Washes were performed with wash buffer using a handheld magnet to prevent loss of magnetic beads in the microparticle cocktail. Biotin antibody cocktail was added to each well and incubated for 1 hour at room temperature with shaking. Wash was repeated and streptavidin-PE was added to each well and incubated for 30 minutes at room temperature before washing. The microparticles were resuspended by adding wash buffer and incubated for 2 minutes with shaking.
