# Supplementary material for: Increased macrophages and changed brain endothelial cell gene expression in the frontal cortex of people with schizophrenia displaying inflammation
Source: Mol Psychiatry. 2018 Sep 13;25(4):761–75. doi: 10.1038/s41380-018-0235-x (PMC7156343; doi:10.1038/s41380-018-0235-x)
Supplement: Supplementary file 2 — Supplementary figure 1 [file 41380_2018_235_MOESM2_ESM.docx]

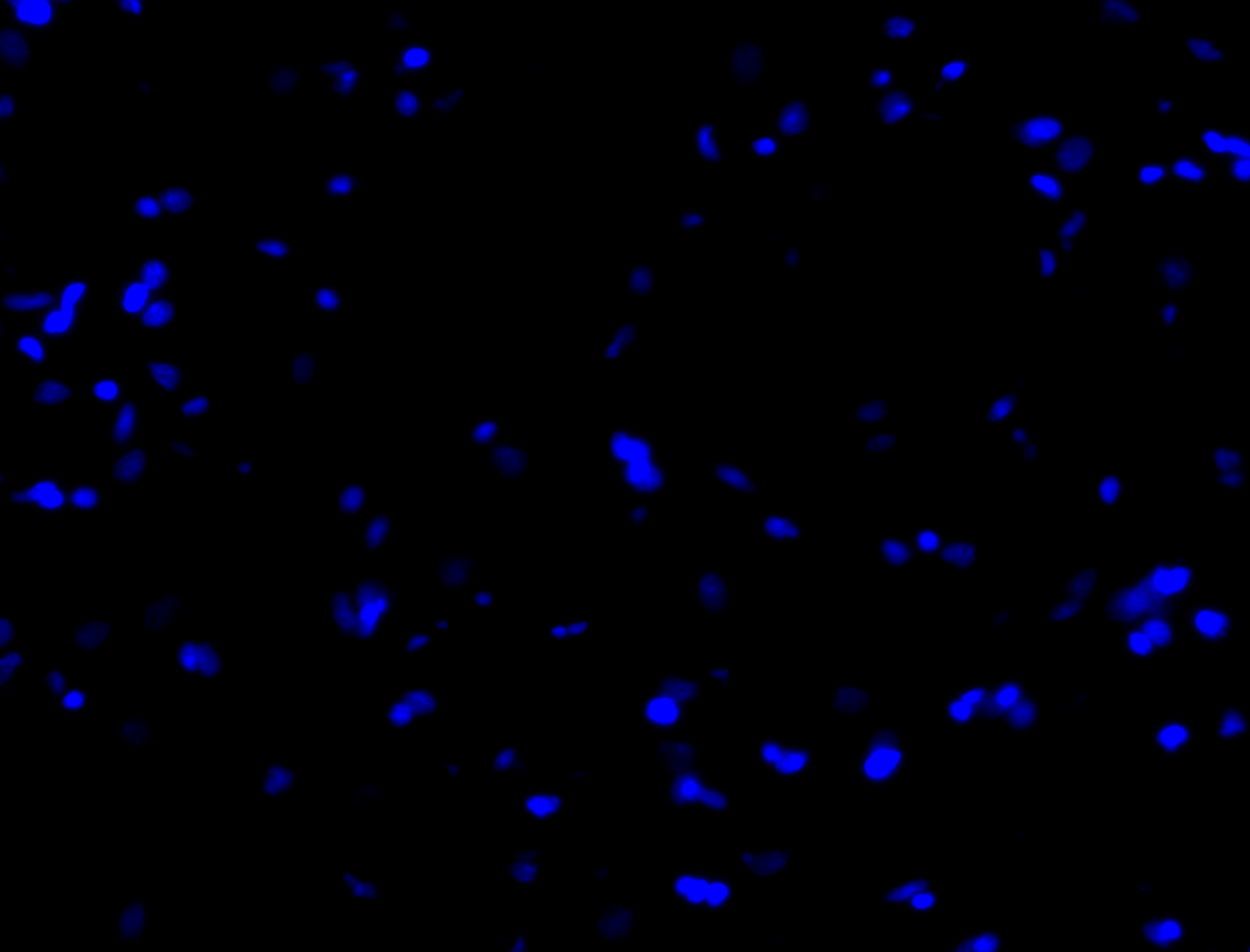


**Supplementary figure 1.** Negative control slide incubated with no primary antibodies, but with secondary antibodies and a DAPI counterstain.
